# Central Skull Base Osteomyelitis in Queensland, Australia, 2010–2020

**DOI:** 10.1093/ofid/ofae614

**Published:** 2024-10-10

**Authors:** Matthew B Eustace, Maxwell Braddick, Kylie Alcorn, Keat Choong, Ferris Satyaputra, David Siebert, Simon Smith, Ryan Sommerville, Kate McCarthy

**Affiliations:** Royal Brisbane Clinical Unit, University of Queensland Faculty of Medicine, Herston, Queensland, Australia; Infectious Diseases Unit, Royal Brisbane and Women's Hospital, Herston, Queensland, Australia; Infectious Diseases Unit, Royal Brisbane and Women's Hospital, Herston, Queensland, Australia; Infectious Diseases Unit, Gold Coast University Hospital, Southport, Queensland, Australia; Infectious Diseases Unit, Sunshine Coast University Hospital, Kawana, Queensland, Australia; Infectious Diseases Unit, Townsville University Hospital, Douglas, Queensland, Australia; Infection Management Services, Princess Alexandra Hospital, Woolloongabba, Queensland, Australia; Infectious Diseases Unit, Cairns Hospital, Cairns, Queensland, Australia; Otolaryngology Unit, Royal Brisbane and Women's Hospital, Herston, Queensland, Australia; Royal Brisbane Clinical Unit, University of Queensland Faculty of Medicine, Herston, Queensland, Australia; Infectious Diseases Unit, Royal Brisbane and Women's Hospital, Herston, Queensland, Australia

**Keywords:** Australia, bacterial infection, fungal infection, osteomyelitis, skull base

## Abstract

**Background:**

Central skull base osteomyelitis (CSBO) is an incompletely defined, life-threatening infection of the bones of the cranial vault. We describe the clinical features and outcomes of CSBO in Queensland, Australia, over an 11-year period.

**Methods:**

Medical record coding enquiries identified cases of CSBO across 6 tertiary hospitals in Queensland, Australia, from January 2010 to December 2020. Epidemiological, demographic, diagnostic, management, and outcome data were collected from each identified case.

**Results:**

Twenty-two cases of CSBO were identified within the study period; the median age was 73 years with a male predominance (73%). High rates of comorbid disease were detected, with a median Charlson Comorbidity Index score of 5. Diabetes mellitus was the most frequently observed condition. Six cases had bone sampling for microbiological diagnosis while the remainder had superficial sampling of contiguous structures. The most common pathogen isolated was *Pseudomonas aeruginosa* followed by *Staphylococcus aureus*, with only 1 case of fungal infection. This series demonstrated a mortality rate of 31.8%, with 45.5% of cases left with long-term sequelae including persistent pain and cranial nerve deficits.

**Conclusions:**

Four key observations emerged in this series: (1) advanced age and diabetes mellitus are common risk factors for CSBO, (2) limited surgical intervention occurred, (3) microbiological diagnoses relied primarily on superficial sampling, and (4) significant mortality and morbidity was observed. Prospective studies are needed to better understand the optimal approach to the diagnosis and management of CSBO and to improve clinical outcomes.

## Teaser

Our case series observed significant mortality and morbidity in cases of central skull base osteomyelitis despite prolonged antimicrobial therapy. Advanced age and diabetes were the most common risk factors. In our jurisdiction, limited surgical intervention for source control occurred and microbiological diagnoses relied on superficial sampling of contiguous, nonsterile sites rather than bone.

## Graphical Abstract

**Graphical Abstract:**
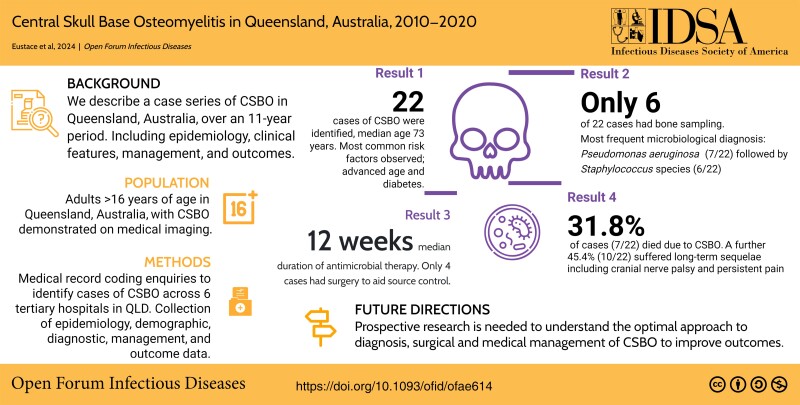
This graphical abstract is also available at Tidbit: https://tidbitapp.io/tidbits/central-skull-base-osteomyelitis-in-queensland-australia-2010-2020

Skull base osteomyelitis (SBO) is a rare, severe, and life-threatening disease that involves infection of the bones of the skull base [1]. Osteomyelitis of the temporal bone secondary to contiguous spread of infection from severe external ear infection, so-called malignant otitis externa (MOE), is a well-recognized entity [2, 3]. In contrast, osteomyelitis primarily involving the clivus, occipital, and sphenoid bones (Figure 1)—described as atypical or central skull base osteomyelitis (CSBO)—is rare and less uniformly defined [4, 5]. CSBO has been described by some authors as SBO involving the central skull base irrespective of source, including as an extension of MOE [1]. Conversely, CSBO has also been defined as SBO involving the clivus, central sphenoid, and occipital bones in the absence of preceding otogenic infection [6]. These cases are often attributed to contiguous spread of sinus, paranasal, and perioral infection [1, 3, 5]. Additionally, individual case reports of CSBO reveal potentially more diverse underlying microbiology compared to typical SBO [5, 7].

**Figure 1.**
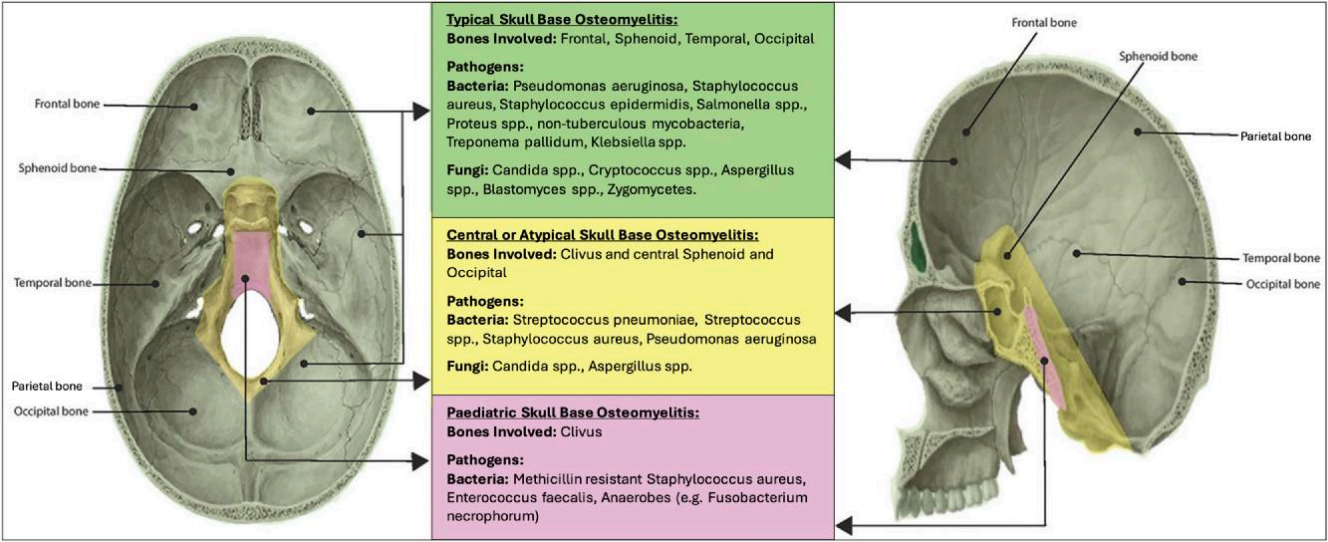
Anatomy of skull base osteomyelitis and its variants. Image adapted from Khan et al [1], “A comprehensive review of skull base osteomyelitis: diagnostic and therapeutic challenges among various presentations,” *Asian Journal of Neurosurgery*, 2018; 13:959–70. This is an open access article distributed under the terms of the Creative Commons Attribution-NonCommercial-ShareAlike 4.0 License (https://creativecommons.org/licenses/by-nc-sa/4.0/). Abbreviation: MRSA, methicillin-resistant *Staphylococcus aureus*.

People with CSBO usually present with features of chronic infection of contiguous structures including the ear, paranasal sinuses, nasopharynx, and oral cavity. Clinical features vary but may include otalgia, nasal congestion, rhinorrhea, oral cavity pain, and headache without improvement despite appropriate loco-regional treatment with procedural intervention, antimicrobials, and anti-inflammatories [4]. As the infection progresses, significant intracranial sequelae may develop including cranial nerve palsies (due to contiguous spread of infection to neural foramina), meningitis, epidural abscess, subdural empyema, brain abscess, and vascular complications (hemorrhage and/or thrombosis) [6, 8, 9]. Without treatment, these intracranial complications invariably lead to significant disability and death.

The diagnosis of CSBO is challenging; clinical history, examination, and laboratory investigations are often variable and nonspecific, with medical imaging essential in establishing the diagnosis [10]. Computed tomography (CT) and contrast-enhanced magnetic resonance imaging (MRI) are the most common modalities used to support the diagnosis of CSBO and to follow progress and response to treatment [3, 11]. Historically, nuclear medicine studies such as gallium-labeled white blood cell scans and technetium-labeled bone scans were utilized for the diagnosis of CSBO. However, with the advent of fluorodeoxyglucose positron emission tomography (PET), these older nuclear medicine modalities have largely been superseded [12]. PET demonstrates excellent sensitivity and greater specificity for the diagnosis of CSBO when compared to MRI and can be a useful adjunct to diagnosis [13].

The microbiological basis of CSBO differs from typical SBO. *Staphylococcus aureus* is the most common causative pathogen, followed by *Pseudomonas aeruginosa* and atypical mycobacteria [14]. CSBO is also more likely to be caused by invasive molds and other fungal pathogens [15, 16]. Due to the rarity of CSBO, the condition remains incompletely defined, with few studies to date and, to our knowledge, none published in the Australian context. Queensland is the northeasternmost state of Australia and spans >7000 km of coastline with a tropical far north region and subtropical southeast region. The state is serviced by a network of public hospitals administered by geographic hospital and health services that receive joint state and federal funding.

This study aimed to describe the clinical features, epidemiology, and outcomes of CSBO in Queensland, Australia, to better understand this rare and life-threatening disease in the Australian context.

## METHODS

A retrospective observational descriptive study of CSBO was undertaken across 6 tertiary hospitals throughout Queensland, Australia, over a 11-year period (1 January 2010–31 December 2020). Cases of CSBO were identified through medical record interrogation via the *International Statistical Classification of Diseases and Related Health Problems, 10th Revision, Australian Modification* definitions [17]. The codes searched included petrositis (H70.2), acute hematogenous osteomyelitis of the skull (M86.08), other acute osteomyelitis of the skull (M86.18), subacute osteomyelitis of the skull (M86.28), chronic multifocal osteomyelitis of the skull (M86.38), chronic osteomyelitis with draining sinus (M86.48), and other chronic hematogenous osteomyelitis of the skull (M86.58) [17].

The medical records of each identified case were reviewed and patients >16 years of age, with medical imaging demonstrating osteomyelitis with bony involvement of 1 or more of clivus, central sphenoid, and central occipital bone, were included. Cases were excluded if an alternative diagnosis accounted for their clinical presentation and imaging findings (eg, base of skull malignancy). Epidemiological data at presentation were collected including patient demographics, comorbidities, presence of predisposing factors (including diabetes, smoking status, and use of injecting drugs), clinical symptoms and signs, presence of cranial nerve palsy, and presumed source of infection (otogenic, paranasal, sinus, hematogenous, other). Investigation data during initial workup, including radiological reports, inflammatory markers, and results of microbiological and histopathological investigations, were also recorded. Finally, data on management and outcomes, including surgical intervention, antimicrobial choice and duration, survival at 12 months, and complications attributable to CSBO, were recorded.

### Patient Consent

The study was approved by the Metro North Health Human Research Ethics Committee A (EC00172) as a low-risk research activity, with the requirement for informed consent waived. The study was conducted in accordance with the Declaration of Helsinki (as revised in 2013).

## RESULTS

We identified 22 cases of CSBO (Supplementary Figure 1) across the 6 participating tertiary centers.

### Demographics and Clinical Presentation

Most cases occurred in men (77.2%) with a median patient age at diagnosis of 73 years (range, 37–96 years) (Table 1). The most common risk factor observed was diabetes mellitus (16/22 [72.7%]) followed by smoking (10/22 [45.5%]), with only a single case, respectively, with a history of injecting drug use or corticosteroid use. The median Charlson Comorbidity Index (CCI) score was 5 (interquartile range [IQR], 2). Frequent presenting symptoms observed included otalgia (7/22 [31.8%]) and headache (7/22 [31.8%]), consistent with the most frequently observed route of infection being otogenic (16/22 [72.7%]). Eight cases had evidence of cranial nerve palsy at presentation, with facial nerve palsy (seventh cranial nerve) seen in 6 of these.

**Table 1.** Demographics and Presenting Features

Case	Gender	Age, y	CCI Score	Risk Factors	Presenting Symptoms	Cranial Nerve Palsy	Route of Infection
1	F	77	5	T2DM, smoking	Nasal discharge	…	Sinus
2	M	73	3	…	Facial pain, otalgia, hearing loss	V3	Paranasal
3	M	89	11	T2DM	Otalgia, vertigo	…	Otogenic
4	M	73	5	T2DM	Headache, dysarthria	…	Otogenic
5	M	55	4	T2DM	Otalgia, tinnitus, hearing loss	…	Otogenic
6	F	62	6	T2DM	Headache, otalgia, nausea, weight loss	…	Otogenic
7	M	37	0	Smoking	Headache, neck pain, otalgia, vertigo	…	Otogenic
8	M	95	8	Smoking	Dysphagia, dysphonia	VII	Otogenic
9	M	74	4	T1DM, smoking	Headache	…	Otogenic
10	M	70	6	T2DM	Headache, fever, weight loss	…	Otogenic
11	M	79	6	Smoking	Facial pain	VII	Otogenic
12	M	87	5	Smoking	Delirium	…	Otogenic
13	M	69	3	T2DM	Diplopia	II, VI	Paranasal
14	M	50	2	T2DM, smoking	Headache	…	Otogenic
15	M	84	7	T2DM, smoking	Hearing loss	VII	Otogenic
16	M	79	4	T2DM, IDU	Neck pain	…	Hematogenous
17	M	53	1	Smoking	Fever, otorrhea, delirium	…	Otogenic
18	M	85	5	T2DM	Facial pain	…	Sinus
19	F	66	4	T2DM	Facial pain, otorrhea	VII	Otogenic
20	F	70	5	T1DM, steroid use	Dysphonia, otorrhea, hearing loss	VII, VIII, IX, X	Otogenic
21	F	96	8	T2DM	Headache, facial pain, otalgia	…	Otogenic
22	M	72	9	T2DM, smoking	Facial pain, otalgia	VII	Otogenic

Abbreviations: CCI, Charlson Comorbidity Index; F, female; IDU, injecting drug use; M, male; T1DM, type 1 diabetes mellitus; T2DM, type 2 diabetes mellitus.

### Laboratory and Radiological Investigations

**Figure 2.**
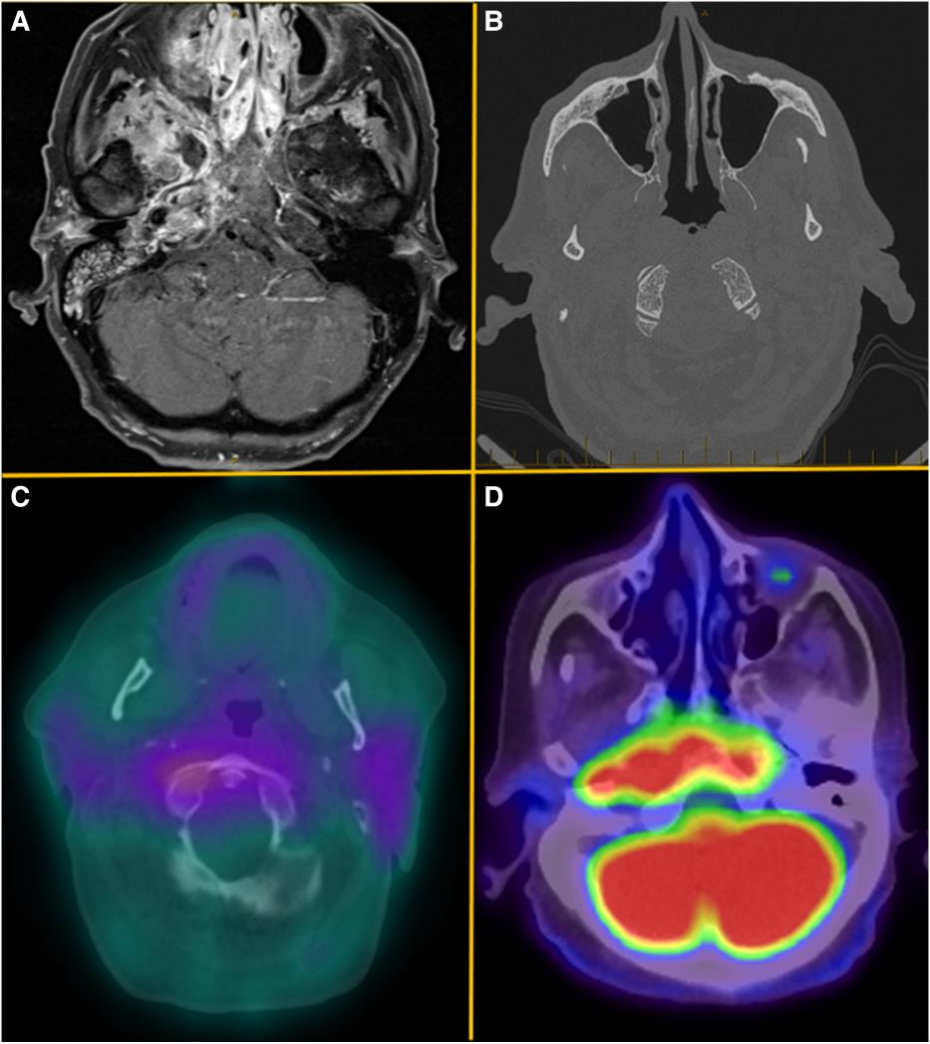
Medical imaging demonstrating features of central skull base osteomyelitis from selected cases. *A* (case 1), Axial T1 gadolinium contrast-enhanced magnetic resonance image demonstrating marked enhancement of paranasal sinuses and right skull base involving clivus, sphenoid, and right petrous apex. *B* (case 9), Axial computed tomographic image demonstrating decortication of the clivus bilaterally along the lateral and anterior margins. *C* (case 4), Axial fused gallium-67 image showing increased gallium-67 tracer uptake extending posteriorly to the region of the clivus. *D* (case 3), Axial fused fluorodeoxyglucose (FDG) positron emission tomography image showing intense FDG uptake eroding the clivus. On the right, FDG uptake extends laterally, eroding the petrous part of the temporal bone and involving the foramens lacerum and jugular foramen.

Laboratory investigations demonstrated a median C-reactive protein of 66 mg/L (IQR, 67 mg/L), erythrocyte sedimentation rate of 65 mm/hour (IQR, 67 mm/hour), and white cell count of 11.6 × 10^9^/L (IQR, 6.85 × 10^9^/L) (Table 2). Thirteen cases had biopsy and histological examination. Features of chronic inflammation and osteomyelitis were observed most frequently. MRI was undertaken in all cases with CT and nuclear medicine studies ordered in 15 and 4 cases, respectively (Figure 2). Bone sampling occurred in 6 cases. The most common microbiological diagnosis was *P aeruginosa* (31.8%), followed by *Staphylococcus* sp (27.3%), with similar findings observed across tropical and subtropical Queensland. Only 1 case of definite fungal CSBO was observed, with *Aspergillus flavus* isolated in a patient residing in tropical North Queensland.

**Table 2.** Laboratory and Radiological Investigations

Case	Peak ESR	Peak CRP	Peak WCC	Imaging	Bones Involved	Biopsy Type	Histology	Sample Type	Microbiology
1	53	26	17	MRI, CT	Temporal, occipital, clivus	…	…	Blood culture	*Staphylococcus aureus*
2	24	2	10	MRI, NM	Temporal, clivus	Endoscopic sphenoidectomy	Acute and chronic inflammation with necrosis in fibrous tissue and bone	Middle ear fluid	*Eikenella corrodens*
3	…	843	9	MRI, CT	Temporal, occipital, clivus	Open biopsy	Nasal lymphoid hyperplasia	Nasal tissue	*Streptococcus agalactiae*, MSF
4	…	29	7	MRI, CT, NM	Temporal, occipital, frontal, clivus	Open biopsy	Acute on chronic inflammation and granulation tissue	Postnasal swab	*Staphylococcus aureus*
5	20	2	14	MRI, CT, NM	Temporal, clivus	…	…	Ear swab	*Pseudomonas aeruginosa*
6	100	35	12	MRI	Temporal, clivus	…	…	Ear swab	*Pseudomonas aeruginosa*
7	34	198	20	MRI, CT	Temporal, clivus	…	…	Blood culture, CSF	*Streptococcus intermedius*
8	…	236	15	MRI, CT	Temporal, clivus	…	…	Ear swab	*Pseudomonas aeruginosa*, MSF
9	133	174	20	MRI	Clivus	CT guided	Chronic inflammation of postnasal space	Postnasal tissue	*Staphylococcus epidermidis*, *Staphylococcus aureus*
10	65	48	14	MRI	Temporal, clivus	Endoscopic sphenoidectomy	Mild chronic inflammation	Ear fluid	*Pseudomonas aeruginosa*, *Staphylococcus epidermidis*
11	75	78	10	MRI	Temporal, occipital	Open biopsy	Bone, connective tissue, and inflammation	Ear tissue	*Pseudomonas aeruginosa*
12	75	67	9	MRI	Temporal, occipital	…	…	Ear swab	Culture negative
13	74	65	11	MRI, CT	Clivus	…	…	Orbital tissue	*Staphylococcus epidermidis*, *Staphylococcus aureus*
14	94	176	26	MRI, CT	Occipital, clivus	Open biopsy	Intense diffuse chronic suppurative inflammation	Mastoid tissue	*Pseudomonas aeruginosa*
15	72	11	12	MRI	Occipital, clivus	…	…	Ear swab	Culture negative
16	55	309	15	MRI, CT	Clivus	…	…	Blood culture	*Staphylococcus aureus*
17	…	248	16	MRI, CT	Occipital, clivus	Endoscopic sphenoidectomy	Chronic inflammation with osteonecrosis	Ear swab	*Streptococcus pneumoniae*
18	43	10	9	MRI, CT	Clivus	Endoscopic sphenoidectomy	Mixed inflammatory cell infiltrate with osteonecrosis, septate fungal hyphae seen	Sinus tissue	*Pseudomonas aeruginosa*, MSF
19	…	109	8	MRI, CT	Temporal, occipital, clivus	Open biopsy	Normal-appearing mucosa	Postnasal tissue	*Pseudomonas aeruginosa*, MSF
20	106	98	21	MRI, CT	Occipital	Open biopsy	Mixed inflammatory cell infiltrate	Postnasal tissue	*Chryseobacterium indologenes*, *Pseudomonas aeruginosa*
21	32	14	6	MRI, CT	Clivus	…	…	…	…
22	121	17	9	MRI, CT, NM	Temporal, clivus	CT guided	Chronic inflammation	Mandibular tissue	*Aspergillus flavus*

Abbreviations: CRP, C-reactive protein (mg/L); CSF, cerebrospinal fluid; CT, computed tomography; ESR, erythrocyte sedimentation rate (mm/hour); MRI, magnetic resonance imaging; MSF, mixed skin flora; NM, nuclear medicine; WCC, white cell count (× 10^9^/L).

### Management and Outcomes

Surgical debridement to support source control occurred in 4 cases; approaches included mastoidectomy (2 cases), endoscopic sinus surgery, and endoscopic infratemporal biopsy and debridement (Table 3). In cases of bacterial CSBO, the median duration of intravenous antimicrobial therapy was 42 days (IQR, 12 days) followed by a median duration of oral antimicrobial therapy of 42 days (IQR, 109.3 days). Seven cases during the study period died due to progressive CSBO, with 3 deaths occurring within 4 weeks of diagnosis. Only 1 case was observed to relapse following treatment completion during the study period, but survived following retreatment. Long-term sequelae of CSBO were observed in 10 cases, including persistent cranial nerve palsy, sensorineural hearing loss, and persistent facial pain.

**Table 3.** Clinical Management and Outcomes

Case	Surgical Intervention	IV Antimicrobials (Duration, d)	PO Antimicrobials (Duration, d)	Relapse	Survival	Complications of Infection
1	…	Cefazolin (3) Flucloxacillin (64) Piperacillin-tazobactam (37)	Dicloxacillin (106) Ciprofloxacin (120) Clindamycin (120)	Y	Y	Right petrous ICA pseudoaneurysm, aspiration
2	FESS	Piperacillin-tazobactam (52)	Amoxicillin-clavulanate (52)	N	Y	Otalgia, hearing loss
3	…	Piperacillin-tazobactam (32)	Co-trimoxazole (28) Ciprofloxacin (89)	N	Y	Nil
4	…	Flucloxacillin (42)	Dicloxacillin (73)	N	Y	Postauricular pain, hearing loss
5	…	Piperacillin-tazobactam (42)	Ciprofloxacin (13)	N	Y	Headaches
6	…	Piperacillin-tazobactam (42)	Ciprofloxacin (42)	N	Y	Tinnitus, hearing loss
7	Right modified radical mastoidectomy	Vancomycin (5) Piperacillin-tazobactam (5) Benzylpenicillin (42) Lincomycin (35)	Amoxicillin (181)	N	Y	Subdural empyema, dural venous sinus thrombosis, IJV thrombosis
8	…	Piperacillin-tazobactam (13)	…	N	N	Death
9	Left mastoidectomy	Vancomycin (33)	Rifampicin (58) Fusidic acid (25)	N	N	Death
10	Endoscopic biopsy infratemporal fossa	Teicoplanin (16) Cefepime (9) Tobramycin (5) Meropenem (61)	…	N	N	Death
11	…	Cefepime (12) Amikacin (29)	…	N	N	Death
12	…	Nil	Ciprofloxacin (23)	N	N	Death
13	…	Ticarcillin-clavulanate (14)	Ciprofloxacin (55) Voriconazole (69)	N	Y	Monocular blindness
14	…	Ceftolozane-tazobactam (75)	…	N	Y	Nil
15	…	Piperacillin-tazobactam (45)	Ciprofloxacin (9) Amoxicillin-clavulanate (9)	N	N	Death
16	…	Flucloxacillin (42)	Cephalexin (42)	N	Y	Neck pain, headaches
17	…	Ceftriaxone (42)	…	N	Y	Nil
18	…	Liposomal amphotericin B (2)	Posaconazole (165)	N	Y	Nil
19	…	Piperacillin-tazobactam (35)	Ciprofloxacin (16)	N	Y	Facial nerve palsy
20	…	Meropenem (10) Vancomycin (10) Piperacillin-tazobactam (42)	Posaconazole (10) Ciprofloxacin (155) Co-trimoxazole (168)	N	Y	Facial nerve palsy, hearing loss
21	…	Piperacillin-tazobactam (42)	Amoxicillin-clavulanate (181) Ciprofloxacin (181)	N	Y	Nil
22	…	Piperacillin-tazobactam (29) Lincomycin (7) Clindamycin (29) Meropenem (15) Cefepime (26)	Voriconazole (193) Posaconazole (16) Ciprofloxacin (162) Amoxicillin-clavulanate (373)	N	N	Death

Abbreviations: FESS, functional endoscopic sinus surgery; ICA, internal carotid artery; IJV, internal jugular vein; IV, intravenous; N, no; PO, oral; Y, yes.

## DISCUSSION

CSBO represents a severe, life-threatening infection with significant risk of morbidity and mortality despite appropriate antimicrobial treatment [6, 9]. This study highlights the challenges in diagnosis and management of CSBO and the detrimental outcomes of death and disability encountered in tropical and subtropical regions of Queensland, Australia. In this case series, 4 key observations emerged—advanced age and diabetes mellitus as risk factors for CSBO, reliance on superficial sampling for microbiological diagnosis, limited surgical intervention offered in this setting, and the significant mortality and morbidity associated with CSBO.

Similar to observational studies of typical SBO, in this series, the most frequently observed comorbidities were advanced age (>65 years) and diabetes mellitus [2]. MOE, a common precursor to SBO, is an infection strongly associated with diabetes, with up to 90% of cases found to have this condition [18]. In our study, patients with CSBO carried a significant burden of comorbidities, categorized as severe by the CCI metric with a median score of 5. Cases were seen in older adults (median age, 73 years) and were highly likely to have diabetes (16/22 [72.7%]); the mechanism for this association between diabetes, advanced age, and CSBO is incompletely understood. However, theories of innate and adaptive immune dysfunction and compromise to vascular supply have been proposed [19]. For example, adaptive immune system effector cell function is demonstrably reduced in diabetes. This is thought to be mediated through impaired antigen-presenting cell function leading to reduced activation of T-helper (Th) 1, Th2, and Th17 cells and thus increased vulnerability to infection, particularly of fungal etiology [20]. While in advanced age, reduced bone marrow hematopoietic potential manifests with reduced production of B cells (especially naive and switch memory B cells), increasing vulnerability to bacterial and viral infections [21]. These mechanisms could all plausibly contribute to the increased frequency of advanced age and diabetes observed in this series, with glycemic control measures offering an important potential adjunct to CSBO management.

Establishing an accurate microbiological diagnosis is crucial in selecting the optimal antimicrobial therapy and can influence the duration of therapy and necessity of surgical debridement (ie, establishing bacterial versus fungal infection) [16]. The microbiological diagnosis of CSBO in most cases in this series was based on superficial or deep sampling of adjacent structures, with only 6 cases obtaining bone samples. In CSBO the most common routes of infection are contiguous spread from nonsterile sites, namely otogenic, paranasal, sinus, and odontogenic [3]. Thus, the absence of representative sampling of involved bone risks isolating a colonizing organism or contaminant rather than the underlying pathogen(s). Within the limitations of this study, it is important to consider that many of the microbiological diagnoses may therefore not have been adequately representative. Thomas described their experience in Vellore, India, where they advocate for biopsy of involved bone to guide antimicrobial treatment in CSBO, particularly in cases with poor response to empiric therapy [22]. Their single center observed additional microbiological yield in deeper surgical samples with no significant complications reported from surgical intervention. This was exemplified in 1 case where an unrecognized fungal co-pathogen was identified via targeted bone biopsy, prompting the addition of antifungal therapy and ultimately resulting in clinical cure [22].

The role of surgery for CSBO is uncertain [6]. Within this series, only 4 cases had surgical intervention involving debridement to aid source control. A further 8 cases had surgical biopsy to aid microbiological diagnosis, though in most cases adjoining structures (ie, paranasal sinus) were sampled, rather than skull base. Mortazavi and colleagues suggest that early recognition and diagnosis of cranial osteomyelitis, followed by aggressive surgical debridement, is essential to achieving good clinical outcomes [7]. Furthermore, early surgical management has been observed to reduce some of the long-term sequelae of infection, that is, persistent facial nerve palsy [8]. Conversely, other centers have observed limited benefit with surgical management but significant procedural risk, routinely managing cases with antimicrobials alone [16, 23]. Compared to bacterial SBO, fungal SBO carries a poorer overall prognosis and consideration of aggressive debridement is strongly advised, especially in cases of zygomycosis [16].

We observed significant morbidity and mortality in this case series with a mortality rate of 31.8% within 12 months due to progressive CSBO. In some cases, acute management was complicated by vascular events (thrombosis, pseudoaneurysm), abscess formation, and aspiration (in the setting of lower cranial nerve deficits). Long-term sequelae were observed in 6 of the surviving cases (27.3%), who had persistent cranial nerve deficits with sensorineural hearing loss, facial nerve palsy, and monocular blindness observed, as well as persistent head and neck pain. The mortality rate observed in this series was greater than other series published in the last 2 decades [16, 24, 25]. We observed a comparatively lower rate of surgical intervention and invasive bone biopsy with many cases not achieving a definitive histological or microbiological diagnosis, limiting directed surgical and antimicrobial management. This may have contributed in part to the observed poor outcomes in this series.

To our knowledge, this is the first case series to report on the epidemiology, investigation, and clinical management of CSBO in Australia. Strengths of this study include the broad geographic area that cases were drawn from, the centralized laboratory information system used by public hospitals, comparison of histological and microbiological data, and the level of detail included around management and outcomes, including antimicrobial selection and duration. However, this study does have some limitations. First, this study was retrospective and observational, based on review of medical records only. Complex case management and multidisciplinary decision making, as are often required in cases of CSBO, may not have been adequately represented or may have been oversimplified. Second, CSBO case identification was based on clinical coding, and not all cases managed across the participating centers may have been captured. Finally, the number of cases over the study period was less than anticipated, resulting in a small sample and limiting the overall generalizability of any findings.

CSBO remains a rare and life-threatening infection. Multidisciplinary collaboration between ear, nose, and throat surgeons, infectious disease physicians, nuclear medicine physicians, and radiologists is paramount to establish an accurate diagnosis and formulate a comprehensive management plan. Significant questions remain over the best approach to confirm the microbiological etiology, the role of surgical debridement, and the optimal antimicrobial duration. Future prospective studies are needed to try and answer these vital questions to broaden understanding and improve clinical outcomes.

## Supplementary Material

ofae614_Supplementary_Data

## Supplementary Data

Supplementary materials are available at *Open Forum Infectious Diseases* online. Consisting of data provided by the authors to benefit the reader, the posted materials are not copyedited and are the sole responsibility of the authors, so questions or comments should be addressed to the corresponding author.

## Note

***Potential conflicts of interest*.** All authors: No reported conflicts.
