# Supplementary figures and images for: Central Skull Base Osteomyelitis in Queensland, Australia, 2010–2020
Source: Open Forum Infect Dis. 2024 Oct 10;11(10):ofae614. doi: 10.1093/ofid/ofae614 (PMC11500440; doi:10.1093/ofid/ofae614)

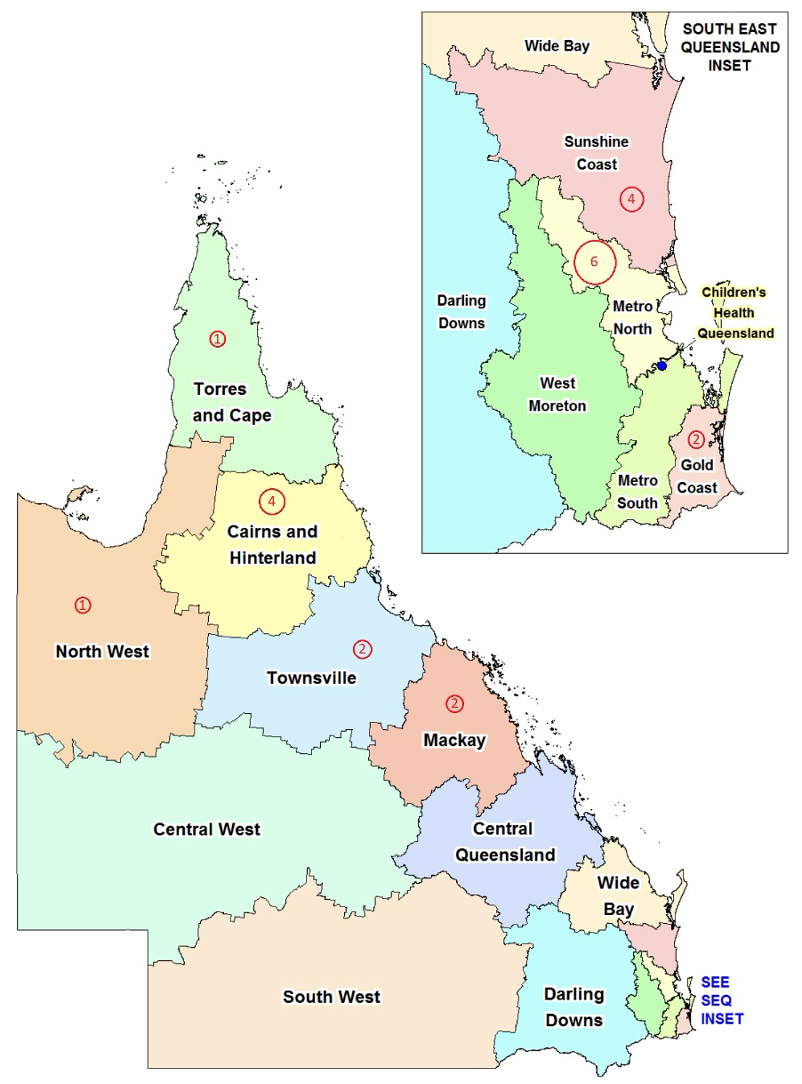

Supplement: ofae614_Supplementary_Data [file ofae614_supplementary_data.zip › Supp Figure 1.png]
